# Post-partum abdominal wall insufficiency syndrome (PPAWIS): lessons learned from a single surgeon’s experience based on 200 cases

**DOI:** 10.1186/s12893-022-01757-y

**Published:** 2022-08-08

**Authors:** Maciej Śmietański, Irmina Anna Śmietańska, Mateusz Zamkowski

**Affiliations:** 1 2nd Department of Radiology, Medical University of Gdansk, Dębinki 7, 80-952 Gdańsk, Poland; 2 Swissmed Hospital in Gdansk, Department of Surgery and Hernia Centre, Gdańsk, Wileńska 44, 80-215 Poland; 3 Department of Anaesthesiology and Intensive Care, Medical University of Gdansk, Gdańsk, Dębinki 7, 80-952 Poland

**Keywords:** PPAWI, PPAWIS, Post partum, Rectus diastasis, Hernia, Abdominoplasty, Abdominal wall, Pregnancy

## Abstract

**Background:**

Post-partum abdominal wall insufficiency (PPAWI) with rectus diastasis is present in over 30% of women after pregnancy. Little is known about how PPAWI affects the social, sexual life and self-esteem of patients. This study was designed to evaluate the safety of onlay mesh combined with abdominoplasty and its impact on the well-being of the patients.

**Method:**

Two hundred patients with PPAWI underwent surgery with onlay mesh and abdominoplasty. The safety of the procedure was assessed by postoperative complications, time of hospitalization and time of drainage. Before the operation and 6 months later, a questionnaire asking about the patient’s sexual and social life and the presence of back pain was completed. The final cosmetic effect was assessed separately.

**Results:**

The onlay procedure with abdominoplasty was found to be safe and fast. The mean operation time was 82 min, and the drainage time was 2.1 days. In this group < 2% postoperative complications were noted. There were no recurrences within the 6 month. Significant improvements in social and sexual life and the level of self-esteem were noted. Back pain was relieved or minimalized in all patients. The final cosmetic effect was insufficient for 2 patients (1%).

**Conclusion:**

PPAWI can be treated safely with onlay mesh and abdominoplasty. The patients’ symptoms were strongly correlated with the morphological status of the front abdominal wall and improved after the procedure. Describing the psychological and social consequences of PPAWI should lead the surgical societies to propose a definition of a new disease called PPAWIS (post-partum abdominal wall insufficiency syndrome).

**Supplementary Information:**

The online version contains supplementary material available at 10.1186/s12893-022-01757-y.

## Background

PPAWI (post-partum abdominal wall insufficiency) is an acronym first used during the Congress of the European Hernia Society in Gdansk in May 2013 by Filip Muysoms. This name is intended to include in one disease entity all symptoms of front abdominal pathology. PPAWI is a pathological condition affecting women after pregnancy characterized by a general laxity of the anterior abdominal wall with diastasis of the rectus abdominis muscles (rectus diastasis - RD), induced by abdominal extension during gestation. This is combined with an excess of skin and panniculus with important repercussions on the general contour of the abdomen and its aesthetics. The resulting instability of the trunk is associated to lumbar back pain and hip pain, which is often noted after pregnancy [1, 2]. However, few studies have shown the impact of PPAWI on quality of life (QoL), and standardized protocols have been used for its assessment. Impact of PPAWI is often measured with use of protocols mainly created to measure quality of life for patients before and after abdominal wall hernia surgery or other psychometric tests (SF-36 {Short Form 36}, EuraHS-QoL { 3-dimensional numerical quality-of-life score introduced by European Registry of Abdominal Wall Hernias}, NHP {Nottingham Health Profile}, BDI {Beck Depression Inventory}, HerQLes { hernia-related quality-of-life survey}, FPFQ {Female pelvic floor questionnaire}). There is lack of dedicated QoL specifically for PPAWI. Available questionnaires are mainly focused on measuring impact of RD of various origins [3–9]. The general shape of the front abdominal wall after pregnancy has aesthetic value, and little is known about how PPAWI influences the psychology and sexual life of women after pregnancy. In many articles, RD or RD combined with midline hernia is treated as a separate disease from a surgical point of view [10, 11]. Involving plastic surgeons in the treatment process to perform a panniculectomy or full abdominoplasty often leads to the incorrect belief that this treatment should only be introduced on patient demand, so medical indications are considered of limited value or do not exist. There are papers strictly focusing on RD and its impact on patient quality of life and social functioning [6–8, 12, 13]. Nevertheless the impact of PPAWI (as a complex psycho-anatomical syndrome) has never been properly described in detail in terms of its effects on social functioning, women’s self-esteem, sexual behaviour and partner-relationship communication problems.

Surgical treatment of RD itself, has been a subject of many publications [10, 11]. The methods include simple plication of the midline (linea alba) to re-approximate the rectus muscle and various mesh techniques. Onlay and sublay techniques have been described as valuable treatment options, but there are few reports about laparoscopic IPOM (intraperitoneal onlay mesh) repair [14, 15]. Descriptions of the methods used often are not discussed in consideration of whether a case of a woman after pregnancy differs from other patients with RD (e.g., obese men, patients after surgical treatment of obesity), and possibly individualized treatment should be expected in this group of patients (women after pregnancy). It seems that while the indications for treatment challenge the decision and there is no consensus on what stage of diastasis should be treated surgically, the type of operation should consider the future decades of well-being and active life of women undergoing surgery [8].

## Methods

### Study design

This study is an assessment of a cohort of 200 women treated for PPAWI with the use of onlay mesh combined with full abdominoplasty, operated on by a single general surgeon over the last five years (2016–2020). The follow-up was designed to assess the value of the surgical technique in a short period (one week to one year after surgery) and to assess the impact of the operation on the social and psychological status of the patients at 6 months after the procedure.

This study was conducted in a hospital in Puck and then at Swissmed Hospital in Gdansk after the author changed working centres (2018). All procedures were performed by general surgeon without any assisting plastic surgeons. The operative technique and meshes used were not changed throughout the study period. A standardized follow-up protocol designed by the author was used for all patients and was conducted by the study secretary (working with the team from 10 years and graduated as medical assistant) during personal meeting. An anaesthesiologist, a leader in pain and behavioural anxiety treatment from the Medical University of Gdansk, was involved as a study team member in all study stages.

Women after a last planned pregnancy were qualified for the surgery during the first visit by surgeon leading the study. The inclusion criteria were as follows: lack of satisfying results after physiotherapy for a minimum of 6 months after delivery (confirmed by the patient), regression of weight to the value expected by the patient, a RD wider than 3 cm with co-existing umbilical hernia or 5 cm with no umbilical defect (measured by callipers), and extended skin with panniculus and/or visible striae gravidarum (pregnancy atrophic linear stretch scars). The exclusion criteria were: obesity (BMI > 30 kg/m^2^), patients’ lack of cooperation and understanding in terms of the “therapeutic purpose” of the surgery (the desire to achieve a good cosmetic effect as an unquestionable priority—such patients were sent to plastic surgeons) and no clear declaration about the end of procreation.

The pathology was described preoperatively with evaluation of the length and width of the RD (measured with US), concomitant hernias, previous operations (mainly C-section), number of pregnancies, skin condition and preoperative back pain. This classification is equal to the classification published in 2019 by the German Hernia Society and International Endohernia Society [16]. However, that publication was issued in 2019, still the concept was discussed widely during various conferences in previous years. A self-made questionnaire assessing social behaviour, sexual activity and communication with the partner was collected before and 6 months after the operation. Questions included in the questionnaire were based on authors experience and collected as the most important and most common from interviews with the patients done before the trial (see Additional file 1). Details of the questions with the results are presented in Tables 1 and 2. It must be mentioned that the questions in both questionnaires are not the same. The point was to describe the pathology and its intense before the operation, and then based on this questions ask modified ones about the grade of improvement after the operation. Still the questions in both questionnaires are paired up to make the statistical conclusions possible (see the questions and possible answers in Tables 1 and 2).

**Table 1:** Self-assessment and back pain before the operation (for 200 included women)

Detected problem	Definitely no	Not as much	I do not know	Yes	Definitely yes
Loss of self-esteem	0 (0%)	0 (0%)	23 (11.5%)	97 (48.5%)	80 (40.0%)
Social life disturbance	3 (1.5%)	10 (5.0%)	11 (5.5%)	90 (45.0%)	87 (43.5%)
Sexual life problems	0 (0%)	7 (3.5%)	23 (11.5%)	51 (25.5%)	119 (59.5%)
Back pain	2 (1.0%)	6 (3.0%)	0 (0%)	171 (85.5%)	21 (10.5%)

**Table 2:** Influence of the surgery on the assessed parameters in the patient’s opinion (for 186 patients after 6 months)

Final cosmetic effect	Bad: I do not wish to see it	Almost bad: I do not accept this	No opinion	Almost good: I accept the scar and shape	Very good: I’m fully satisfied	p value
	0 (0%)	2 (1.1%)	1 (0.6%)	28 (15.0%)	155 (83.3%)	Not applicable
Did the operation change your self-esteem?	Definitely no	Rather no	I don’t know	Rather yes	Definitely yes	
	0 (0%)	0 (0%)	1 (0.6%)	34 (18.3%)	151 (81.1%)	p < 0.05
Did the operation change your social life?	Definitely no	Rather no	I don’t know	Rather yes	Definitely yes	
	0 (0%)	3 (1.6%)	0 (0%)	33 (17.8%)	150 (80.6%)	p < 0.05
Did the operation improve your sexual life?	Definitely no	Rather no	I don’t know	Rather yes	Definitely yes	
	0 (0%)	7 (3.8%)	2 (1.1%)	18 (9.6%)	159 (85.5%)	p < 0.05
Did the operation resolve your back pain problems?	Definitely no	Rather no	I don’t know	Rather yes	Definitely yes	
	0 (0%)	0 (0%)	9 (4,8%)	49 (26.2%)	128 (68.8%)	p < 0.05

From the surgical perspective, a standardized European Hernia Society protocol was used [17]. The primary outcome was seroma formation and time of needed drainage of the subcutaneous tissue, secondary presence of infection, haematoma or other surgical site complications in one to three weeks after operation. In the long-term follow-up of min. 6 month, recurrence and skin dysmorphia (e.g., skin laxity, “dog ears”, umbilical dysmorphia) were noted. The questionnaire was conducted before and 6 month after the surgery.

Data are presented as the mean and range for numerical data and as percentages for categorical values for answers from the questionnaire. Microsoft Excel software was used to collect and analyse the data.

### Operative technique

The operation was performed by the general surgeon only. However, training by the qualified plastic surgeon was conducted for 2 month before the start of study. Trials have shown positive results with onlay mesh for incisional hernia prevention with a low rate of seroma and other wound complications [18, 19]. Based on this assumption, the onlay mesh position was chosen for this study.

The incision is extensive and is typically made from ASIS (Anterior Superior Iliac Spine) to ASIS along the natural suprapubic crease. The scar after caesarean section (if present) is removed. A plane between the fascia and fatty tissue with subcutaneous flap formation is then created superiorly up to the costal margin until the xiphoid process is reached. Laterally, at the level of the xiphoid process, the preparation reaches the mammary line. The umbilicus is circumferentially dissected from the flap, leaving it attached to the abdomen by the umbilical stalk and fat. Special care is taken to leave enough of a fat pad circumferentially around the umbilicus to avoid ischaemia and necrosis, as the plexus surrounding the umbilical stalk contains its blood supply. The medial margins of the anterior rectus sheaths are plicated (single plication) with continuous long-lasting absorbable suture (PDS 2−0) to approximate the muscles with inversion of a widened linea alba to avoid subcutaneous ridge formation. This suture allows also the umbilical hernia defect closure. Flat macroporous polypropylene mesh (Optilene Mesh, BBraun Asculap, Tuttlingen, Germany, pores 1.5 mm, weight 60 g/m^2^) covering the midline (width of 3 cm) was implanted and fixed with two nonabsorbable continuous sutures on the lateral edges of the mesh (polypropylene 3−0) to the fascia (anterior rectus sheath). Mesh size and margin used for the surgery are based on previous mathematical and biomechanical studies published by our team [20–23].

After careful measurement, the marked excess skin and fat are excised and the cranial flap is reapproximated to the lower incision in multiple suture layers to strengthen the closure and avoid tension on the skin layer. The umbilicus is then implanted into the flap with interrupted skin sutures (4−0). Two drains are applied vertically in the flanks. Finally, the skin flap is sutured with continuous short absorbable sutures (4−0). An abdominal binder is applied at the end of the procedure.

Abdominoplasty can be performed differently in cases of minimal skin changes and a location of the umbilicus over half of the length of the midline (high position). The umbilicus is cut from the fascia, moved down with the skin flap and fixed again to the middle line ca. 2–3 cm below the previous location.

### Follow-up period

Postoperative data were collected according to the German Hernia Society protocol [16]. Medical records from the hospital were collected, including the time of drainage, amount of collected fluids, haematoma, SSI and possible mesh infection. The first follow-up visit was conducted 7 days after the operation (first follow-up visit or time of suture removal). The presence of seroma (US examination), haematoma or infection was noted, and the pain score and return to daily activity were questioned and noted. After 6 months, a questionnaire was conducted via phone or personal visit by the study secretary. At the same time pictures of the front abdominal wall was sent by the patient to the study secretary to assess the cosmetic result, knowing that phone call and digital solutions are reliable and safe follow-up instruments [24, 25]. The pictures (3 perspectives—front, lateral and 45^o^ oblique) were assessed/compared with preoperative pictures by the surgeon involved only in follow-up stage of study.

## Results

Two hundred consecutive patients were included in the study. The follow-ups were completed by 100% of the patients 7 days after the operation and 93% (14 patients were lost to contact) after 6 months.

The age of the patients varied from 29 to 45 years (mean 35). Various numbers of pregnancies were noted from only one to five (mean 2). 85% of the assessed group had a history of at least one C-section.

Table 3 presents the morphological changes of the front abdominal wall. It must be mentioned that the majority of the patients presented with advanced morphological changes with the presence of an umbilical hernia and large midline dilatation.

**Table 3:** Status of the front abdominal wall before the operation (for 200 included women)

skin	No striae gravidarum	Striae gravidarum around naval not changing the belly shape	As before with pursing	Striae gravidarum and pursing with the change of the shape	Striae gravidarum and skin flaps
	37 (18.5%)	32 (16.0%)	76 (38.0%)	42 (21.0%)	13 (6.5%)
Linea alba	Less than 3 cm	3.0–4.9 cm	5.0–6.9 cm	7.0–9.0 cm	More than 9 cm
	17 (8.5%)	27 (13.5%)	131 (75.5%)	22 (11.0%)	3 (1.5%)
Umbilical hernia	No hernia	Less than 1 cm with preperitoneal fat tissue	1–2 cm	up to 3 cm with hernia sac	Over 3 cm
	9 (4.5%)	158 (79.0%)	23 (11.5%)	7 (3.5%)	3 (1.5%)

The average operation time was 82 min (range 63–115, mean 82). In the early postoperative period, 3 haematomas (one needed reoperation for evacuation of the haematoma) were noted. Twelve seromas (thickness less than 10 mm) were noted. Two seromas needed evacuation. One needed drainage at 2 weeks. The other was evacuated 4 times and was finally resolved after 6 weeks. The mean postoperative drainage time was 2.1 days (range from 1 to 7 days), and the aggregate volume of fluid collected was 93 mL (range from 50 to 260 mL). No surgical site infections (SSI) were noted. The postoperative surgical data are summarized in Table 4. All cases of additional operations (i.e., correction of scar folds) were performed approximately 3 months after the primary surgery. In 23 patients, correction of the lateral incisional scar folds (so-called “dog ears”) was performed under local or short intravenous anaesthesia in ambulatory settings. In one case, the shape of the umbilicus (an asymmetric scar fold) was corrected.

**Table 4:** Demographic data, intra- and postoperative data for the chosen values (values for 200 women, recurrence after 6 months for 186)

BMI (kg/m^2^) Pregnancies (number) Age (years) Recti diastasi (size, cm) Operation time (minutes) Time of hospitalization (days) Time of drainage (days) Drainage fluid collected (mL)	22.6 (19.2–26.8) 2 (range: 2–5) 35 (range: 29–45) 7.6 (range: 6–12) 82 (range: 63–115) 2.1 (range: 1–6) 2.1 (range: 1−7) 93 (range: 50–260)
Postoperative complications (n)	
Seroma (needed intervention) Haematoma (needed intervention) Margin skin necrosis Partial umbilicus ischaemia SSI (Surgical Site Infection) RD recurrence (after 6 month) Additional re-operation (“dog ears”, umbilicus dysmorphia)	5/2 (2.5%/1%) 3 / 1 (1.5%/0.5%) 1 (0.5%) 1 (0.5%) 0 0 23 (12.36%)

A questionnaire about social and sexual functioning was conducted 6 months after the operation and 93% of the women completed the follow-up. The results of the questionnaire are summarized in Table 1. It must be noted that 2 patients did not appreciate the cosmetic effect of the operation. In both cases, extreme skin folds (striae) were present. Even after abdominoplasty, the condition of the skin was poor and hardly acceptable to the patients, but the shape and function were judged as improved. In all cases, the operation allowed for back pain relief or significant improvement. Additionally, the improvement of sexual and social functioning was noted in the majority of cases. All statistical analysis of the results were based on repeated measures (Friedman tests). p < 0.05 was considered as a statistically significant difference (Table 2).

## Discussion

### Key results and interpretation

According to results, PPAWI is a combination of morphological changes with the inclusion of back pain and social functioning disorders affecting a significant proportion of women after pregnancy. Sperstad in a large study of Norwegian women found that mild diastasis (2–3 fingers, ca. 5–6 cm) was found in 31% of women one year after delivery, and in 1%, the diastasis was moderate or severe (over 3 fingers) [26]. Interestingly, in that study, none of the studied factors describing the pregnancy (age, height, weight and weight gain, baby’s birth weight or delivery mode) influenced the prevalence of RD. Other previous studies have shown similar results [27]. Surgical treatment of RD does not have a consensus and options varies from simple suture plication to endoscopic or robot-assisted procedures and no approach shows superiority over others [11, 28, 29]. Only double plication of diastasis has showed lower complication rate, still not increasing recurrence rate. The guidelines of the European and Americas Hernia Society for patients with RD and small concomitant umbilical or epigastric hernia recommended mesh placement instead of suture repair based on the high risk of recurrence in the sutured group [30]. This recommendation has been given; however, it was not focused on PPAWI, and the quality of evidence is low. Although the Guidelines do not provide the solution to the questions, it is very important remark there is no consensus about treatment of RD and tailored approach and shared-decision process between patient and surgeon must be done [31].

The choice of the technique in our study was based on various assumptions, which includes the fact that the subject of this non-life saving procedure are women with a long life expectancy. It forces to leave peritoneal cavity intact to prevent possible adhesions and not having negative impact on the function of the trunk. Prior IPOM publications showed the opposite, therefore IPOM procedure was abandoned [32, 33].

The sublay technique requires opening the rectus sheath and involves a posterior layer of the rectus muscle in the inflammatory process of mesh ingrowth. In the past, onlay mesh was associated with seroma formation, but recently published data with the use of macroporous monofilament meshes did not confirm these findings [30]. Knowing that thinning and stretching of the linea alba is an important risk factor for actual development of midline hernias (umbilical, epigastric, trocar, incisional hernia) due to the deterioration of the connective tissue and the pulling of the abdominal muscles [16] and to compare the procedure with other surgical techniques, the mesh is used for the prevention of future RD recurrences and hernia formation.

In this paper, the authors assessed the value of the onlay mesh approach with full abdominoplasty and its impact on physical functioning and social and sexual life, as well as on the self-esteem of the treated women. We demonstrated that simultaneous single midline plication with onlay mesh and abdominoplasty is a safe, feasible, fast, and in our opinion, less tissue damaging method for PPAWI treatment. It should be recognized that there are no long-term results comparing onlay and retromuscular mesh in women with PPAWI or IPOM or endoscopic techniques versus open ones. On the other hand, the follow-up protocols are concentrated on widely accepted end points of the studies, including short-term complications such as seroma or infection and long-term recurrence. The authors believe that tailored approach should be considered when non life-saving procedures in women after pregnancy are proposed to improve their shape and quality of life.

We did find only few previously published papers describing psychological, sexual and social problems in the context of belly deformation or about the possible impact of the operation on these aspects of life, although they were focused on the impact of RD of various origin alone [1, 3–9]. Olsson showed significant improvement in the SF-36 questionnaire one year after surgery and Temel showed that operation reduces significantly depression signs assed with BDI scores [6, 9].

#### Limitations and strength of this study

This study has limitations. The inclusion criteria were restricted to symptomatic PPAWI and patients looking for surgical intervention, so the results may not be applicable to all PPAWI cases. The patients were qualified for surgery a minimum of 6 months after delivery, but it was not checked in the protocol if they had had sufficient physiotherapy before the surgery, simply accepting the patient statement. The protocol assessed only a few areas of human well-being because it was based on interviews with PPAWI patients. That is why other potentially important areas of mental health could be missing in this study. The protocol is also not validated to the general population of women after delivery. It is based on authors experience and does not have any similar ones in the literature. We have decided to apply this protocol in the study to show the importance of psycho-sexual factors on the well-being of described women population. We found that protocols validated for measuring impact of RD or hernia operations does not fully apply in this specific situation. That is why authors believe that creating and conducting fresh questionnaire for newly described phenomena is a strong element of this study.

On the other hand, this study was performed by an experienced team, and the surgical procedure was performed by one surgeon with over 20 years of experience in large ventral hernia repair. Compared to the other published studies on this topic, it is based on a relatively large heterogeneous group of patients, which makes its findings more valuable. It has also been proven that there is no necessity for the presence of plastic surgeons during the operation. In our opinion personal training in the procedure showed to be sufficient for general surgeon. The study team included female secretaries, and the anaesthesiologists were also women, which possibly bridges the divide in the interview touching on personal areas of the patient. To our knowledge, this is the first study assessing the impact of surgery on chosen behavioural areas in women’s lives. In light of this study, it must be pointed out that PPAWI is a complex pathology not expected as a consequence of pregnancy but resulting in long-lasting deformation. Struggling to raise a child while suffering from a loss of self-esteem leads to a decreased quality of life and social problems. Findings from this study shows that morphological changes (extended skin with panniculus and/or visible striae gravidarum) and coexisting small hernia (umbilical or linea albae) are also key problem in women after pregnancy. In the questionnaire for psychological assessment, most of the women reported a strong influence of PPAWI on back pain, social functioning, self-esteem and sexual life (Table 3). In the descriptive assessment (collected as comments), the most frequently noted problems were at the beach or swimming pool, the necessity of changing the style of dressing (can only wear sac-like clothing), being treated as pregnant again (e.g., on public transport or in the office) and stress during sexual activity independent of the type of partner (in a long-term relationship or a brand new one). Another conclusion coming from this study shows that umbilical hernia is present in most cases and should also be included in the description of PPAWI. This also revalidate what Kohler et al. previously stated [34].

### Generalizability

PPAWI is not only a morphological pathology but also or even mainly a psychological disorder leading to social exclusion. It must also be noted that in many countries (including the authors’ country of origin), asking for treatment for PPAWI is shamed and thought of as a whim. Describing the psychological and social consequences of PPAWI should lead the surgical societies to propose a definition of a new disease called PPAWIS (post-partum abdominal wall insufficiency syndrome). Further study should be conducted with the participation of psychologists and sexologists to gain more extended knowledge about the phenomena. Changing the definition (from PPAWI to PPAWIS) can also lead to different perceptions of the problem. We should appreciate how much a simple and safe procedure can change the life of thousands of patients. In the majority of the human population, discussions about the need for treatment can make it easier for women, while science will deliver arguments for it.

## Supplementary Information

**Additional file 1**. Patients questionnaire.

## Data Availability

The datasets used and/or analysed during the current study are available from the corresponding author. All data is in Polish language but it can be translated and presented on reasonable request.
