# Supplementary material for: Post-partum abdominal wall insufficiency syndrome (PPAWIS): lessons learned from a single surgeon’s experience based on 200 cases
Source: BMC Surg. 2022 Aug 8;22:305. doi: 10.1186/s12893-022-01757-y (PMC9358894; doi:10.1186/s12893-022-01757-y)
Supplement: Supplementary file 1 — Additional file 1. Patients questionnaire. [file 12893_2022_1757_MOESM1_ESM.docx]

**PPAWIS QUESTIONAIREE No……………………..**

1. Patients name:
2. Age:
3. Number of labors (amount, c-section, twins?)

………………………………………………………………………………………………………………………………………………………

1. Operation date:

**Status of the front abdominal wall before the surgery (Mark X)**

| **Skin** | No striae gravidarum | Striae gravidarum around naval not changing the belly shape | As before with pursing | Striae gravidaum and pursing with the change of shape | Striae gravidaum and skin flaps |
| --- | --- | --- | --- | --- | --- |
| **Linea alba** | Less than 3 cm | 3-4,9 cm | 5-6,9 cm | 7-9 cm | More than 9 cm |
| **Umbilical hernia** | No hernia | Less than 1cm with preperitoneal fat tissue | 1-2 cm | Up to 3 cm with hernia sac | Over 3 cm |

SELF ASSESSMENT QUESTIONAIREE BEFORE THE SURGERY .

| **Loss of self-esteem** | Definitely no | Not as much | I do not know | Yes | Definitely yes |
| --- | --- | --- | --- | --- | --- |
| **Social life disturbance** | Definitely no | Not as much | I do not know | Yes | Definitely yes |
| **Sexual life problems** | Definitely no | Not as much | I do not know | Yes | Definitely yes |
| **Back pain** | Definitely no | Not as much | I do not know | Yes | Definitely yes |

**Belly button transposition (Mark X)**

Belly button transfer to flat ---

No belly button transfer ----

Surgical complications ---

Complication type ………………………………………………………………………………………………………………..

Dog-ears re-plasty (yes/no)…………………… (months after surgery)………..

AFTER SURGERY QUESTIONAIREE

Date ……………………………..

| **Cosmetic effect** | Bad – I do not wish to see it | Almost bad – I do not accept this | No opinion | Almost good – I accept the scar and shape | Very good – I’m fully satisfied |
| --- | --- | --- | --- | --- | --- |
| **Did the operation change your** self-esteem**?** | Definitely no | Rather no | I don’t know | Rather yes | Definitely yes |
| **Did the operation change your social life?** | Definitely no | Rather no | I don’t know | Rather yes | Definitely yes |
| **Did the operation improve your sexual life?** | Definitely no | Rather no | I don’t know | Rather yes | Definitely yes |
| **Did the operation resolve your back pain problems?** | Definitely no | Rather no | I don’t know | Rather yes | Definitely yes |
